# Description of an Epidemic of Dengue

**Published:** 1877-01

**Authors:** James W. Hereford

**Affiliations:** Mercer’s Bottom, West Virginia


Description of an Epidemic of Dengue.—*By James W. Hereford, M.D., of Mercer's Bottom, West Virginia.*—An unprecedented flood in the Ohio river, during the month of August, 1875, destroyed thousands of acres of green corn, and other crops, in this vicinity, which, being mixed with the sediment deposited by the receding river, and all left exposed to the action of the intensely hot weather which immediately followed, produced a most sickening stench, and impressed every one with the idea that some dreadful pestilence would result therefrom. Contrary, however, to all our predictions, the fall and winter of 1875 passed away without any great increase in the number or malignancy of our ordinary diseases, and it was not until the previously overflowed lands had been broken for the spring crop of 1876 that anything occurred directly traceable to the flood of August, 1875.

About this time, intermittent fever, dumb ague, masked chill, malarial roseola, splenitis, and other malarial phenomena, began to be of frequent occurrence, all of which were accompanied by more than the usual amount of bone-ache, and required double or treble the quantity of quinine to break them up and prevent a recurrence than such diseases usually do.

Finally, on the 1st of May, 1876, I encountered the first well-marked case of what, during the late war, I saw a great deal of in the South, under the name of dengue, or breakbone fever. This case may be regarded as a fair average, in severity and duration, of the whole number of cases which fell under my observation during the past summer. I will therefore give it in detail.

The number of cases treated during the summer was twenty-two.

Case I. May 1st, 1876, I was called to see J. G., a farmer, aged thirty years, supposed to be laboring under acute rheumatism. He seemed to be suffering the most intense agony I had ever witnessed in the human subject, occasionally becoming so frantic that it required several attendants to keep him in bed. His own description was that his bones were being ground up in a bark-mill, and his joints crushed by a blacksmith's vise. On inquiry I found that the paroxysm came on regularly at ten o'clock every morning, and continued without any mitigation until four o'clock the next morning. A slight remission would then occur, lasting until ten o'clock, as before mentioned. No swelling, no tenderness, or other indication of arthritic inflammation was present. The bowels were constipated, the tongue much swollen, and with a foul, pasty coat extending clear under its tip. No appetite for food. Stomach very irritable. Skin bathed in perspiration. Body temperature very high, but extremities cold as marble. Pulse 120, full and strong. Urinated very little at a time, and that with great difficulty. His mind was clear. He had not slept a moment since first attacked, three days previous to my visit. As I arrived near the time of the expected remission, I concluded to remain until that event occurred. In the meantime I attempted to administer chloroform by inhalation, and at first with some degree of success, but owing to the irritable condition of the stomach I was obliged to desist, as it seemed to produce the most distressing nausea. At four o'clock he appeared somewhat easier. Pulse 100. Extremities beginning to warm up, which had been encouraged by the use of jugs of hot water to the feet, and mustard cataplasms to the wrists and ankles. The following was prescribed:

℞.—Quinæ Sulphatis, . . . . . ʒss

Hydrargyri Submuriatis, . . . gr.xv. M.

For three powders. One to be taken at four o'clock, one at seven o'clock, and the last at nine o'clock. If the paroxysm recurred, he was to take:

℞.—Pulveris Doveri, . . . . . ʒij

Hydrargyri Submuriatis, . - . gr.xij. M.

For twelve powders. One to be given every hour, until the pains were relieved. At midnight he was to take an ounce of olei ricini, with twenty drops of olei terebinthinœ, in hot whisky toddy. As a local application, the following was directed to be frequently applied.

℞.—Tincturæ Aconitæ,

Tincturæ Opii,

Tincturæ Camphoræ,

Chloroformi, . . . . . . aa ʒj. M.

For the difficult micturition, he wrs ordered a tablespoonful, every hours, of the following mixture:

℞.—Sodœ Carbonatis, . . . . . ʒiij

Spiritus nitratis dulcis, . . . . ʒj

Aquæ menthæ piperitæ, . . . ʒv. M.

The whole plan of treatment was strictly adhered to, and had the effect of prolonging the remission until two o'clock P.M., at which time the pain and fever again occurred, with unmitigated severity. The opiate was given as directed, but with very little relief. The oil and turpentine given at midnight operated by four o'clock, and as the remission came on at this time, he felt much better. This day he had ten-grain doses of quinine every three hours until six doses were given. Paroxysm postponed to nine o'clock in the evening, when it returned, and continued on through the night with apparently greater intensity than ever, although he had a grain of sulphate of morphia every hour during the night, given under my own personal supervision. A drachm and a half of quinine was given the next day, which shortened the paroxysm an hour or so, but did not lessen the intensity of it, while it was on, a particle. This night I had the patient immersed in a barrel of water as hot as could be borne, and allowed him to remain in it twenty-five minutes. He was then rolled up in blankets, and put to bed, and soon broke out in a profuse perspiration, after which he slept well until morning, his first sleep since the beginning of the attack. An eruption made its appearance this morning, which very much resembled that of rubeola. This I thought was a final solution of the whole matter. I thought that the obstinacy of the disease was owing to the tardy appearance of the eruption. The tongue had lost its foul, pasty coat, and assumed a smooth, silvery or scalded appearance;. the tip and edges still fiery red. Although there was now a profuse diarrhœa, I thought his general condition was somewhat better, as his appetite had improved a little, and his suffering was not so great. The quinia, in five-grain doses, combined with the same quantity of Dover's powder, was given steadily through the day, at intervals of two hours. The paroxysm appeared several hours earlier to-day than usual, and lasted all night without a single interval of rest. The hot bath was again resorted to, but it absolutely seemed to aggravate his sufferings· He had opiates almost *ad libitum.*

As I had become nearly as desperate as my patient, and thinking that the non-action of the medicines might depend upon their not being absorbed when taken into the stomach, and not having a hypodermic syringe, I had several small fly blisters drawn over the most painful parts, and at one time sprinkled a grain of sulphate of morphia on the denuded surface, but no effect whatever toward mitigating the pain was produced by this method.

In addition to large quantities of morphine, given as before stated, he took by inhalation four ounces of sulphuric ether during the night, but without any effect toward relief. Opiates, given in any quantity consistent with the safety of the patient, I consider utterly useless. I have kept the pupil of the eye contracted to the size of a pin point for hours and days together, without any stupefying effect. My patient up to this time had taken over a half ounce of quinine, without its ever having produced its characteristic effect on the brain. Conceiving the idea that the firmly contracted state of the pupil, caused by the action of the opiates, in some inexplicable manner interfered with the action of the quinine, I concluded to substitute belladonna, an agent that, if given in sufficient quantity, would allay pain and dilate the pupil. I immediately put my conception into practice. He was ordered a grain of the extract of belladonna every four hours, alternated with ten grains of quinine; and I soon had the satisfaction of witnessing the peculiar effect of the mydriatic, followed by the loud complaint from my patient that his ears were ringing as if all the bells of London were turned loose in his head at once.

From this time on all was clear sailing. The paroxysms of pain became lighter each day, under the influence of the belladonna and quinine, until the night of the eighteenth day of the attack was passed without a single twinge of pain. He was now placed on small doses of quinine and carbonate of iron, but his convalescence was extremely slow and tedious. The extensor muscles of the lower extremities were entirely paralyzed, and the general prostration was extreme. He began to improve under the use of an elixir of quinine, iron and strychnia; and at this time, October 14th, he has almost entirely regained his usual health and strength.

Of my other cases, all I have to say is, that they were of every grade of severity, from even worse than the case just related down to cases so mild as to be with difficulty recognized. Fortunately, most of them came in after I had the experience gained by the treatment of my first case, and of course had the benefit of it. I lost but one case out of twenty-two treated, and that a child of three years of age, which died in convulsions, with the eruption fully out.—*Med. and Surg. Reporter.*

